# CX3CL1 and IL-15 Promote CD8 T cell chemoattraction in HIV and in atherosclerosis

**DOI:** 10.1371/journal.ppat.1008885

**Published:** 2020-09-25

**Authors:** Soumya Panigrahi, Bonnie Chen, Mike Fang, Daria Potashnikova, Alexey A. Komissarov, Anna Lebedeva, Gillian M. Michaelson, Jonathan M. Wyrick, Stephen R. Morris, Scott F. Sieg, Mirko Paiardini, Francois J. Villinger, Karem Harth, Vikram S. Kashyap, Mark J. Cameron, Cheryl M. Cameron, Elena Vasilieva, Leonid Margolis, Souheil-Antoine Younes, Nicholas T. Funderburg, David A. Zidar, Michael M. Lederman, Michael L. Freeman

**Affiliations:** 1 Center for AIDS Research, Division of Infectious Diseases and HIV Medicine, Department of Medicine, Case Western Reserve University School of Medicine/University Hospitals, Cleveland Medical Center, Cleveland, OH, United States of America; 2 Department of Population and Quantitative Health Sciences, Case Western Reserve University School of Medicine, Cleveland, OH, United States of America; 3 Laboratory of Atherothrombosis, Moscow State University of Medicine and Dentistry, Moscow, Russia; 4 Department of Cell Biology and Histology, School of Biology, Moscow State University, Moscow, Russia; 5 Cleveland Louis Stokes Veterans Affairs Medical Center, Cleveland, OH, United States of America; 6 Division of Microbiology and Immunology, Yerkes National Primate Research Center, and Department of Pathology and Laboratory Medicine, Emory University School of Medicine, Atlanta, GA, United States of America; 7 New Iberia Research Center, University of Louisiana at Lafayette, New Iberia, LA, United States of America; 8 Harrington Heart & Vascular Institute, University Hospitals, Cleveland Medical Center/Case Western Reserve University, School of Medicine, Cleveland, OH, United States of America; 9 Department of Nutrition, Case Western Reserve University School of Medicine, Cleveland, OH, United States of America; 10 Section on Intercellular Interactions, Eunice Kennedy Shriver National Institute of Child Health and Human Development, National Institutes of Health, Bethesda, MD, United States of America; 11 School of Health and Rehabilitation Sciences, Ohio State University, Columbus, OH, United States of America; Vaccine Research Center, UNITED STATES

## Abstract

Atherosclerotic cardiovascular disease (ASCVD) remains an important cause of morbidity in the general population and risk for ASCVD is increased approximately 2-fold in persons living with HIV infection (PLWH). This risk is linked to elevated CD8 T cell counts that are abundant in atherosclerotic plaques and have been implicated in disease pathogenesis yet the mechanisms driving T cell recruitment to and activation within plaques are poorly defined. Here we investigated the role of CD8 T cells in atherosclerosis in a non-human primate model of HIV infection and in the HIV-uninfected elderly; we sought to identify factors that promote the activation, function, and recruitment to endothelium of CX3CR1+ CD8 T cells. We measured elevated expression of CX3CL1 and IL-15, and increased CD8 T cell numbers in the aortas of rhesus macaques infected with SIV or SHIV, and demonstrated similar findings in atherosclerotic vessels of HIV-uninfected humans. We found that recombinant TNF enhanced the production and release of CX3CL1 and bioactive IL-15 from aortic endothelial cells, but not from aortic smooth muscle cells. IL-15 in turn promoted CX3CR1 surface expression on and TNF synthesis by CD8 T cells, and IL-15-treated CD8 T cells exhibited enhanced CX3CL1-dependent chemoattraction toward endothelial cells *in vitro*. Finally, we show that CD8 T cells in human atherosclerotic plaques have an activated, resident phenotype consistent with *in vivo* IL-15 and CX3CL1 exposure. In this report, we define a novel model of CD8 T cell involvement in atherosclerosis whereby CX3CL1 and IL-15 operate in tandem within the vascular endothelium to promote infiltration by activated CX3CR1+ memory CD8 T cells that drive further endothelial activation via TNF. We propose that these interactions are prevalent in aging and in PLWH, populations where circulating activated CX3CR1+ CD8 T cell numbers are often expanded.

## Author summary

People living with HIV infection and elderly HIV-uninfected persons have increased risk of developing atherosclerotic cardiovascular disease, and have increased numbers and/or proportions of CD8 T cells that express the vascular endothelium-homing receptor CX3CR1. Atherosclerotic plaques contain many activated CD8 T cells, which have been implicated in disease pathogenesis, yet the mechanisms driving T cell recruitment to and activation within plaques are not clear. Here we propose a model in which CX3CR1+ CD8 T cells promote endothelial dysfunction by the combined effects of CX3CL1, IL-15, and TNF. Persistent inflammation triggers endothelial cell activation and dysfunction in people living with HIV infection. Endothelial cell-derived CX3CL1 then directs the migration of CX3CR1+ T cells to the activated endothelium where IL-15 activates T cells to express TNF. TNF drives endothelial expression of CX3CL1 and IL-15, providing a feed-forward loop of activation. We provide evidence that these pathways are active in human atherosclerotic plaques and in the aortic endothelium of SIV/SHIV-infected rhesus macaques. We propose these mechanisms of T cell-induced endothelial damage are operative in traditional risk factor-associated atherosclerosis in the general population and are accelerated in people with HIV infection who live in a state of sustained chronic inflammation.

## Introduction

Combination antiretroviral therapy (ART) has dramatically increased the survival of persons living with HIV infection (PLWH), but this prolonged lifespan is accompanied by increased risk of atherosclerotic cardiovascular disease (CVD) that is an important cause of morbidity in the elderly general population [1–4]. Atherosclerosis is an immunologic, inflammatory disease yet the intercellular interactions that lead to plaque development and severity are not well characterized. Defining pathways that promote atherosclerosis is critical to identifying novel targets for prevention and treatment in PLWH and in the general aging population.

Cardiovascular morbidity in PLWH on ART is linked to an expansion of effector CD8 T cells in circulation [5]. Many of the expanded CD8 T cells express high levels of the vascular-endothelium homing chemokine receptor CX3CR1, and plasma levels of its ligand–fractalkine (CX3CL1)–are upregulated in HIV infection and in atherosclerosis [6–8]. CX3CR1 and CX3CL1 contribute to CVD morbidity in persons without HIV infection: polymorphisms in *CX3CR1* are associated with coronary artery disease [9, 10]; numbers of CX3CR1-expressing cells and plasma CX3CL1 levels predict plaque rupture in unstable angina [11, 12]. We have recently demonstrated that CD8 T cells and CD68+ myeloid cells co-localize at sites of endothelial dysfunction in aortas of SIV and simian-human immunodeficiency virus (SHIV)-infected rhesus macaques (RM) [13], and that atherosclerotic plaques from HIV uninfected persons are enriched for activated CD8 T cells [14]. Activated CX3CR1+ CD8 T cells are potent cytokine producers [7, 8], and T cell-derived TNF can promote expression of the procoagulant tissue factor on monocytes [15]. CX3CL1 expression by dysfunctional endothelium and/or smooth muscle could provide a mechanism to attract and bind effector CX3CR1+ CD8 T cells and this may be important in the initiation and progression of atherosclerosis in both PLWH and in HIV-uninfected persons. We hypothesize that migration of CX3CR1+ CD8 T cells into plaque could contribute to endothelial damage in atherosclerosis through direct targeting of activated endothelium and/or indirect cytokine-mediated activity, possibly by activating other infiltrating inflammatory cells [13, 15].

Interleukin (IL)-15, which has been detected within atherosclerotic plaques [16, 17] and contributes to atherogenesis in a mouse model [18], is a strong activator of CD8 T cells, inducing expression of cytolytic molecules and enhancing cytolytic potential [19, 20]. As IL-15 governs the proper trafficking of CD8 T cells to peripheral tissue sites [21–24], we hypothesize that IL-15 serves a central role in the recruitment and activation of CD8 T cells in atherosclerosis, and that local expression of CX3CL1 and IL-15 work in concert to induce the activation and chemoattraction of CX3CR1+ CD8 T cells that are capable of endothelial damage, in turn promoting development of atherosclerosis.

Here we propose that IL-15 drives a pro-chemotactic phenotype on CD8 T cells that are then primed for endothelium localization via CX3CR1 ligation. Once CD8 T cells are in close proximity to endothelial cells (ECs), they induce EC activation and dysfunction at least in part via elaboration of TNF. We show here that ECs (but not smooth muscle cells) activated by TNF express CX3CL1 and bioactive IL-15, which promote CD8 T cell migration in a CX3CL1/CX3CR1-dependent manner. Thus, we define a pro-atherosclerotic feed forward loop that may drive atherosclerotic CVD in PLWH and in aging. Supporting our model, we demonstrate increased expression of CX3CL1 and IL-15 in human atherosclerotic plaques and in the aortic endothelium of SIV/SHIV-infected RM, reflecting microenvironments that sustain endothelial dysfunction. Furthermore, our finding that CD8 T cells within human plaques are both activated and have reduced CX3CR1 expression underscores the relevance of this mechanism *in vivo*.

## Methods

### Ethics statement

All human experiments were approved by the Institutional Review Boards of University Hospitals Cleveland Medical Center, or Moscow State University of Medicine and Dentistry. Participants provided written informed consent, in accordance with the Declaration of Helsinki. All animal experiments were approved by the Institutional Animal Care and Use Committee of Yerkes National Primate Research Center.

### Human donors and tissues

Peripheral blood was acquired from HIV-uninfected controls and from PLWH on combination ART with undetectable plasma HIV RNA (<40 copies/mL). Characteristics of blood donors are presented in S1 Table and atherosclerotic plaque donors are presented in S2 Table. Human vascular tissue sections, including from carotid arteries, were purchased from US Biomax. Atherosclerotic plaque tissue was acquired in sterile saline following clinically indicated carotid or femoral endarterectomy procedures, then preserved for sectioning or processed to derive a single-cell suspension, by digestion with 0.2mg/ml DNase I (Roche) and either 0.016mg/ml Liberase (Roche) or 1.25mg/ml Collagenase XI by shaking at 250rpm for 1h at 37°C, straining through a 40μm nylon cell filter (BD), and washing with PBS.

### Animals and infection

Descending thoracic aorta sections were obtained from 16 RMs (female n = 7; male n = 9) infected with [SIVmac239smr (n = 10), SHIVSF162P3 (n = 4), or SHIV 2873 (n = 2)], and 16 uninfected female RM. Clinical information on these RMs has been presented previously [13].

### Cell culture

Primary human aortic endothelial cells (HAoECs) and smooth muscle cells (HAoSMCs) (PromoCell) were cultured in Endothelium Cell Growth Medium MV (PromoCell) or Smooth Muscle Cell Growth Medium 2 (PromoCell), respectively, supplemented with antibiotic antimycotic solution (Millipore-Sigma) and treated for 1, 2, 4, and 7 days with recombinant human TNF (210-TA; R&D Systems) or recombinant human IL-15 (247-ILB; R&D Systems). Relative expression of CX3CL1, IL-15, IL-15Rα and CCL2 was measured by fluorescence microscopy, real-time PCR (Taqman Gene Expression Assays; ThermoFisher), or in supernatant by ELISA (R&D Systems).

### Histology and immunostaining

For immunostaining, cells and deparaffinized sections were incubated overnight at 4°C with antibodies to human CX3CL1 (goat polyclonal; R&D Systems), human IL-15 (either mouse monoclonal clone #34559; R&D Systems, or rabbit polyclonal; Abcam), and/or IL-15Rα (rabbit monoclonal; Abcam), followed by staining with AlexaFluor488- or Cy3-conjugated anti-goat, anti-mouse, and anti-rabbit secondary antibodies (Life Technologies) as appropriate and mounted in Vectashield with DAPI (Vector Laboratories) for fluorescence microscopy. To detect viral proteins, RM tissues were stained with anti-SIVmac p27/Gag (55-2F12; from Dr. Niels Pedersen [cat# 1610] obtained through the NIH AIDS Reagent Program, Division of AIDS, NIAID, NIH [25]), followed by staining with pre-adsorbed Cy3-conjugated goat anti-mouse IgG (Life Technologies). Sections of RM aorta or carotid atherosclerotic plaques were imaged by fluorescence microscopy and analyzed using ImageJ software (NIH), with iterative deconvolution methods (up to 10 iterations) to enhance and study high-resolution images. Target to nuclear fluorescence ratio (Fc/n) was determined according to the formula: Fc/n = (Fc-Fb)/(Fn-Fb), where Fb is background auto-fluorescence [26].

### Flow cytometry

T cell phenotype was assessed by flow cytometry (LSRFortessa, BD) using fluorochrome-conjugated antibodies specific for CD3 (clone UCHT1; BD), CD4 (SK3; BD), CD8 (SK1; BD), CD45RO (UCHL1; BD), CX3CR1 (2A9-1; BioLegend), CD57 (HNK-1; BioLegend), CD27 (M-T271; BD), CD28 (CD28.2; BioLegend), CD69 (FN50; BD), and CCR7 (3D12; BD). Viable cells were gated using Live/Dead Aqua viability dye (Invitrogen). For induction and detection of intracellular cytokines, cells were cultured overnight with 20ng/ml recombinant human IL-15 (247-ILB; R&D Systems) or medium control (RPMI 1640 [Gibco], supplemented with 10% fetal bovine serum [Gemini Bio-Products], 1% L-glutamine [Gibco], and 1% penicillin/streptomycin [Gibco]), then treated with brefeldin A (GolgiPlug, BD) for 6h prior to harvest. After Live/Dead and surface staining, cells were fixed and permeabilized with Cytofix/Cytoperm (BD) for 20 min on ice and stained for 40 minutes on ice with anti-IFNγ (B27, BD) and anti-TNF (MAb11, BD). For intracellular accumulation of cytolytic molecules, cells were treated with IL-15-supplemented or control medium for 48 hours, then harvested, stained with Live/Dead and surface antibodies, treated with Cytofix/Cytoperm, stained with anti-granzyme B (GB11; BD) and anti-perforin (B-D48; BioLegend).

### Purified T cell stimulation assays

AutoMACS (human pan T cell isolation kit, Miltenyi Biotec)-purified T cells were labeled with CellTrace Violet (Molecular Probes) then treated for 1, 2, 4, or 7 days with CX3CL1 (100ng/ml) or IL-15 (20ng/ml). After treatment, T cells were immunostained and analyzed by flow cytometry, and supernatant TNF was measured by ELISA (Quantikine TNF ELISA; R&D Systems).

### RNA-sequencing

CD8 T cells were purified from peripheral blood of HIV-uninfected donors (n = 5) by AutoMACS separation (human CD8+ T cell isolation kit, Miltenyi Biotec). Following stimulation with IL-15 (20ng/ml) or RPMI 1640 medium control for 24h, RNA was harvested using the RNeasy Plus kit (Qiagen), and RNA-Seq libraries were prepared with TruSeq Stranded Total RNA kits (Illumina), then sequenced using an Illumina NextSeq550 High Output flowcell. Differential expression analysis was performed using EdgeR and LIMMA packages, and gene set enrichment was performed using the GSVA package in R (Bioconductor).

### Chemoattraction assay

HAoECs were cultured with TNF (10ng/ml) or medium for 7 days. Purified T cells were that had been pre-stimulated with IL-15 (20ng/ml) or medium control for 2 days were then added to the cultures on the seventh day, separated from HAoECs by a 5μm collagen-coated transwell membrane (Corning). In some assays, T cells were pre-treated with 500nM AZD8797 (Axon MedChem) for 1h prior to placement in the upper transwell chamber. T cells from the upper and lower chambers were harvested separately 3h later, and examined by flow cytometry. Absolute cell counts from the upper and lower chambers were determined using Liquid Counting Beads (BD).

### Statistics

Comparisons between unrelated groups used nonparametric two-tailed Mann Whitney U tests. Comparisons among three or more groups were performed with nonparametric Kruskal-Wall tests with Dunn’s multiple comparison post-tests. Paired group analyses used Wilcoxon matched-pairs signed rank test. All statistics were performed using Prism (GraphPad). Differences were considered statistically significant if the P-value was less than 0.05.

### Data and materials availability

RNA-Seq data are submitted to the Gene Expression Omnibus and Sequencing Read Archive (GSE154644).

## Results

### Aortic endothelium in SIV/SHIV infection is enriched for CX3CL1 and IL-15 expression

**Fig 1:**
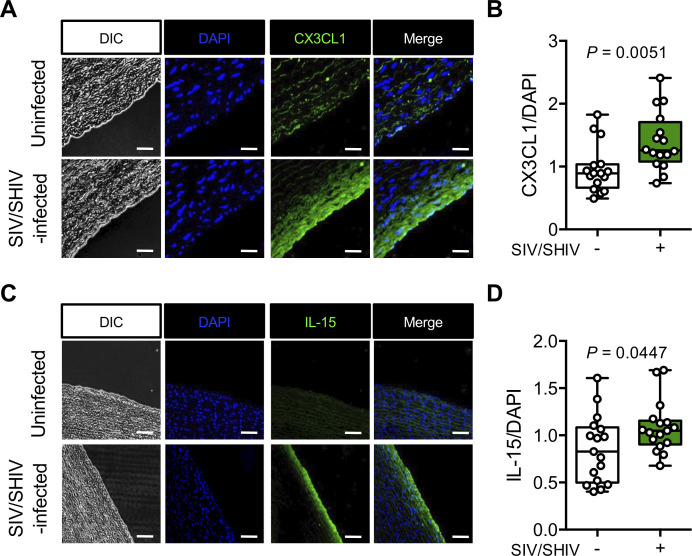
CX3CL1 and IL-15 levels are elevated in aortas from SIV/SHIV-infected RMs. **(A,C)** Endothelial DAPI and CX3CL1 **(A)** or IL-15 **(C)** levels in uninfected controls (n = 16; upper row) and from SIV/SHIV-infected RMs (n = 16; lower row). **(B,D)** Ratio of CX3CL1 **(B)** or IL-15 **(D)** fluorescence to DAPI fluorescence per animal (n = 16), calculated from ~300 RM aortic endothelial cells per sample. Differences between groups were determined by Mann-Whitney U test. DIC, differential interference contrast.

We previously reported increased infiltration of CD8 T cells and CD68+ myeloid cells (monocytes/macrophages) at sites of endothelial dysfunction in aortas of RMs with SHIV and SIV infections [13]. Here we show that expression of both CX3CL1 (Fig 1A and 1B) and IL-15 (Fig 1C and 1D) was greater in the aortas of SHIV/SIV-infected animals than in the aortas of uninfected controls (IgG control staining shown in S1A Fig), suggesting a possible role for CX3CL1 and IL-15 in the recruitment of immune cells to inflamed vasculature. Interestingly, among the infected monkeys, aortas from male animals contained significantly more CX3CL1 than aortas from female animals, whereas IL-15 expression was not different (S1B Fig). We could also detect p27/Gag in aorta sections from SIV/SHIV-infected animals but not from uninfected animals (S1C Fig), indicating that cells containing SIV/SHIV are present in vascular tissue and may contribute to endothelial dysfunction.

### CX3CL1 and IL-15 expression is enriched in human atherosclerotic lesions

**Fig 2:**
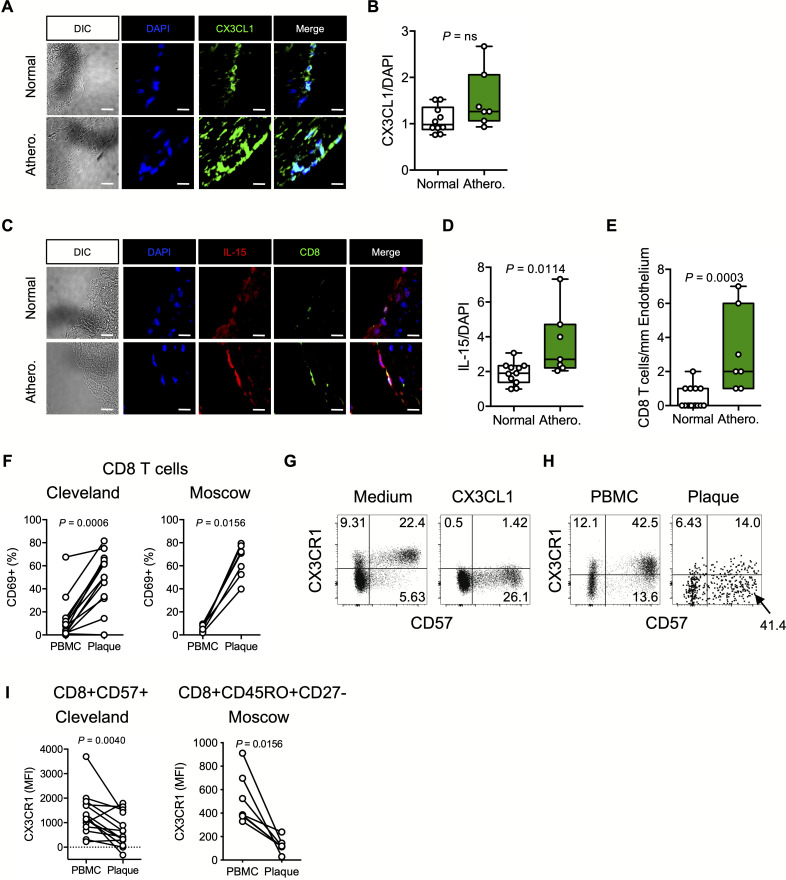
Expression of CX3CL1 and IL-15 in human atherosclerotic plaque tissue in proximity to CD8 T cells. **(A)** Endothelial DAPI and CX3CL1 expression in artery sections from normal donors (n = 10, top) and from donors with atherosclerosis (n = 7, bottom). **(B)** Ratio of CX3CL1 fluorescence to DAPI fluorescence per section. **(C)** Endothelial DAPI and IL-15 expression in artery sections from normal donors (n = 10, top) and from donors with atherosclerosis (n = 7, bottom). **(D)** Ratio of IL-15 fluorescence to DAPI fluorescence per section. **(E)** Number of CD8 T cells per mm endothelium within sections from normal donors (n = 15) and from donors with atherosclerosis (n = 7). Differences between groups were determined by Mann-Whitney U test. **(F)** Proportion of CD8 T cells that express CD69 in PBMCs or donor-matched plaque tissues from donors from Cleveland, Ohio, USA (n = 13) and Moscow, Russia (n = 7). **(G)** Representative dotplots (n = 2) showing surface expression of CX3CR1 and CD57 on peripheral blood CD8 T cells following overnight exposure to CX3CL1 or medium control. **(H)** Representative dotplots (n = 13) showing surface CX3CR1 and CD57 expression on CD8 T cells derived from the plaque and peripheral blood. **(I)** Surface CX3CR1 MFI on CD57+ CD8 T cells from donors from Cleveland, Ohio, USA (n = 13, left) and on effector memory (CD45RO+CD27-) CD8 T cells within PBMCs or donor-matched plaque tissue from donors from Moscow, Russia (n = 7, right). Differences between groups were determined by Wilcoxon rank sum test. DIC, differential interference contrast; PBMC, peripheral blood mononuclear cells.

We next measured CX3CL1, IL-15, and CD8 T cells in carotid tissues from HIV-uninfected donors with or without atherosclerosis. There was a trend toward more CX3CL1 expression (Fig 2A and 2B), significantly greater IL-15 expression (Fig 2C and 2D), and a significant increase in CD8 T cells (Fig 2E) in atherosclerotic tissues. Additionally, within plaques (S2A Fig), we found robust CX3CL1 and IL-15 protein expression, in some cases proximate to infiltrating CD8 T cells (S2B Fig). Consistent with previous reports of activated CD8 T cells within plaques [14, 27, 28], we found that when compared to circulating CD8 T cells from the same donors, plaque-infiltrating CD8 T cells (gating strategy shown in S2C Fig) had robust expression of CD69 (Fig 2F, left), an activation molecule that promotes tissue residency by binding sphingosine-1-phosphate receptor 1 (S1PR1) [29–31]. Increased CD69 expression was also found on plaque-infiltrating CD8 T cells in a separate cohort of endarterectomy donors from Moscow, Russia (Fig 2F, right). Peripheral blood CD8 T cell expression of CX3CR1 was significantly positively correlated with age in our cohorts of HIV-uninfected plaque donors (S1D Fig). To determine if plaque-infiltrating CD8 T cells had been recently exposed to CX3CL1, we examined CD57+ CD8 T cells, which are predominantly CX3CR1+ in circulation and downregulate CX3CR1 upon CX3CL1 ligation (Fig 2G). We have recently demonstrated that CD57+ CD8 T cells are enriched for cytolytic granules and cytokine production, thereby possibly contributing to endothelial dysfunction [32]. Plaque CD57+ CD8 T cells had significantly lower CX3CR1 surface expression than autologous CD57+ CD8 T cells in peripheral blood (Fig 2H and 2I). This difference was also seen with plaque and blood effector memory CD8 T cells in the Moscow cohort (Fig 2I), suggesting that plaque-derived CD57+ CD8 T cells may have been recently exposed to CX3CL1 which we propose targets them to the plaque *in vivo*. We recently observed similar evidence for activation and CX3CL1 exposure in plaque-infiltrating CD4 T cells [33].

### Differential expression of CX3CL1 and IL-15/IL-15Rα by human aortic endothelial cells (HAoECs) and human aortic smooth muscle cells (HAoSMCs)

**Fig 3:**
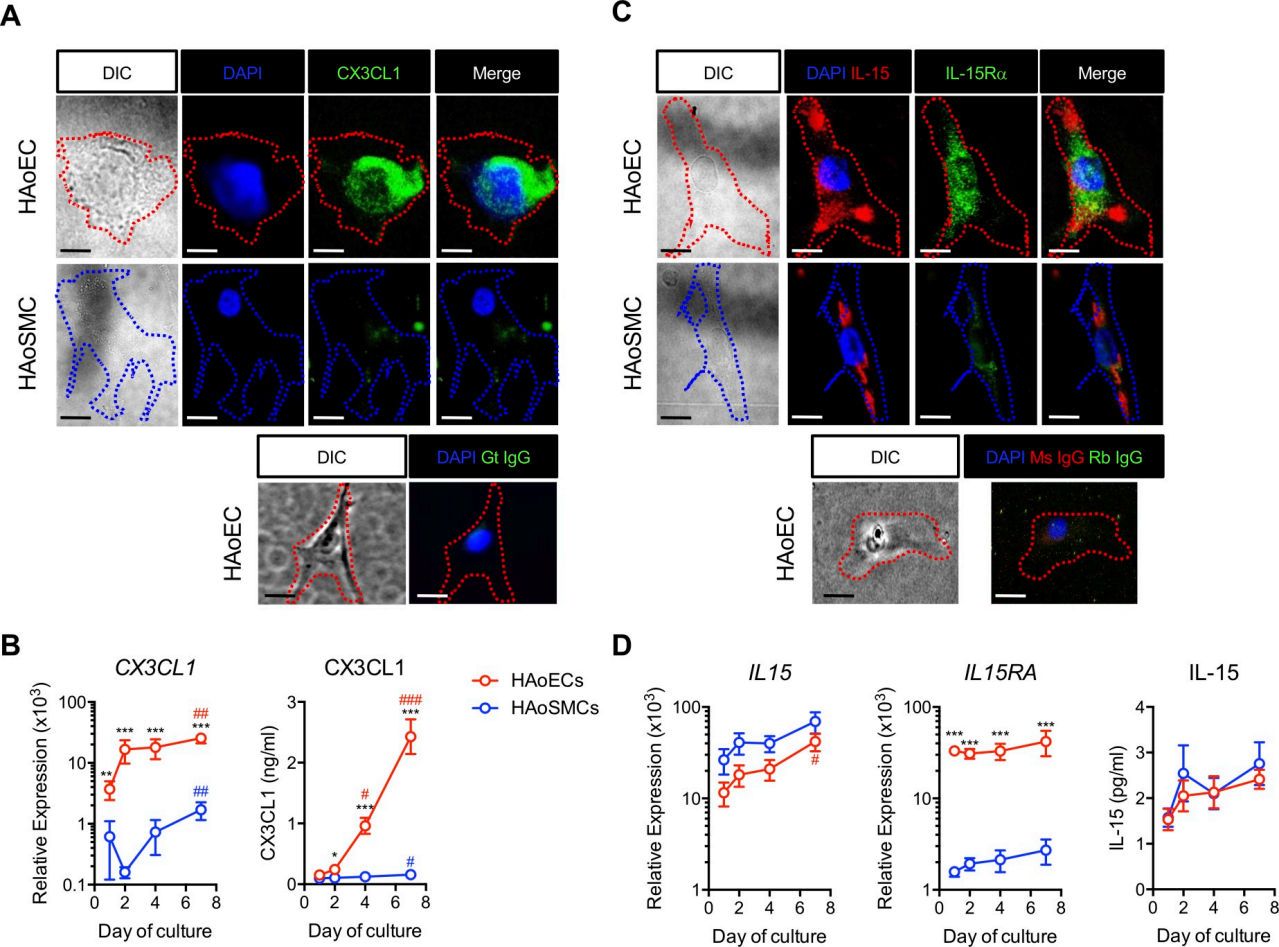
Differential expression of CX3CL1 and IL-15/IL-15Rα by HAoECs and HAoSMCs. **(A)** CX3CL1 protein expression in HAoEC (upper row) and HAoSMC (lower row). **(B)** Relative *CX3CL1* expression compared to GAPDH (left) and CX3CL1 secretion (right) in HAoECs (n = 8) and HAoSMCs (n = 8). **(C)** Constitutive surface expression of IL-15 and IL-15Rα in HAoEC (upper row), and in HAoSMC (lower row). **(D)** Relative expression of *IL15* and *IL-15RA* compared to GAPDH (left and middle) and IL-15 secretion (right) in HAoECs (n = 7) and HAoSMCs (n = 7). **(B,D)** Data are mean ± SEM. Differences between groups were determined by Mann-Whitney U test: *P ≤ 0.05; **P ≤ 0.01; ***P ≤ 0.001. Differences within groups were determined by Kruskal-Wallis test with Dunn’s correction: #*P* ≤ 0.05; ##*P* ≤ 0.01; ###*P* ≤ 0.001. HAoECs, human aortic endothelial cells; HAoSMCs, human aortic smooth muscle cells; DIC, differential interference contrast; Gt, goat; Ms, mouse; Rb, rabbit.

To characterize the vascular cell types that have the potential to engage and activate circulating T cells, we examined expression of CX3CL1, IL-15, and the α-chain of the IL-15 receptor (IL-15Rα) in cultured primary HAoECs and HAoSMCs. We detected expression of CX3CL1 on HAoECs but relatively low expression on HAoSMC surfaces (Fig 3A). Consistently, *CX3CL1* gene expression was significantly greater in HAoECs than in HAoSMCs (Fig 3B, left), and only HAoECs secreted CX3CL1 protein (Fig 3B, right).

Surface IL-15 was detected on both HAoEC and HAoSMC, but IL-15Rα was detected on only HAoECs (Fig 3C), and expression of *IL15* and *IL15RA* RNAs followed the same pattern (Fig 3D). These findings are significant because the bioactive form of IL-15 is *trans*-presented as a heterodimer complexed with IL-15Rα [34, 35]. HAoECs and HAoSMCs secreted low, but equivalent, levels of IL-15 protein in culture (Fig 3D, right), but this assay only weakly identifies IL-15 in its heterodimeric form. Therefore, while both HAoECs and HAoSMCs have the potential to supply IL-15, only HAoECs have the capacity to *trans*-present it in its bioactive form and are the most likely source of CX3CL1 in the vascular microenvironment with potential to interact with CX3CR1+ CD8 T cells.

### IL-15 drives CD8 T cell proliferation, activation, and an inflammatory, vascular homing, cytolytic phenotype

**Fig 4:**
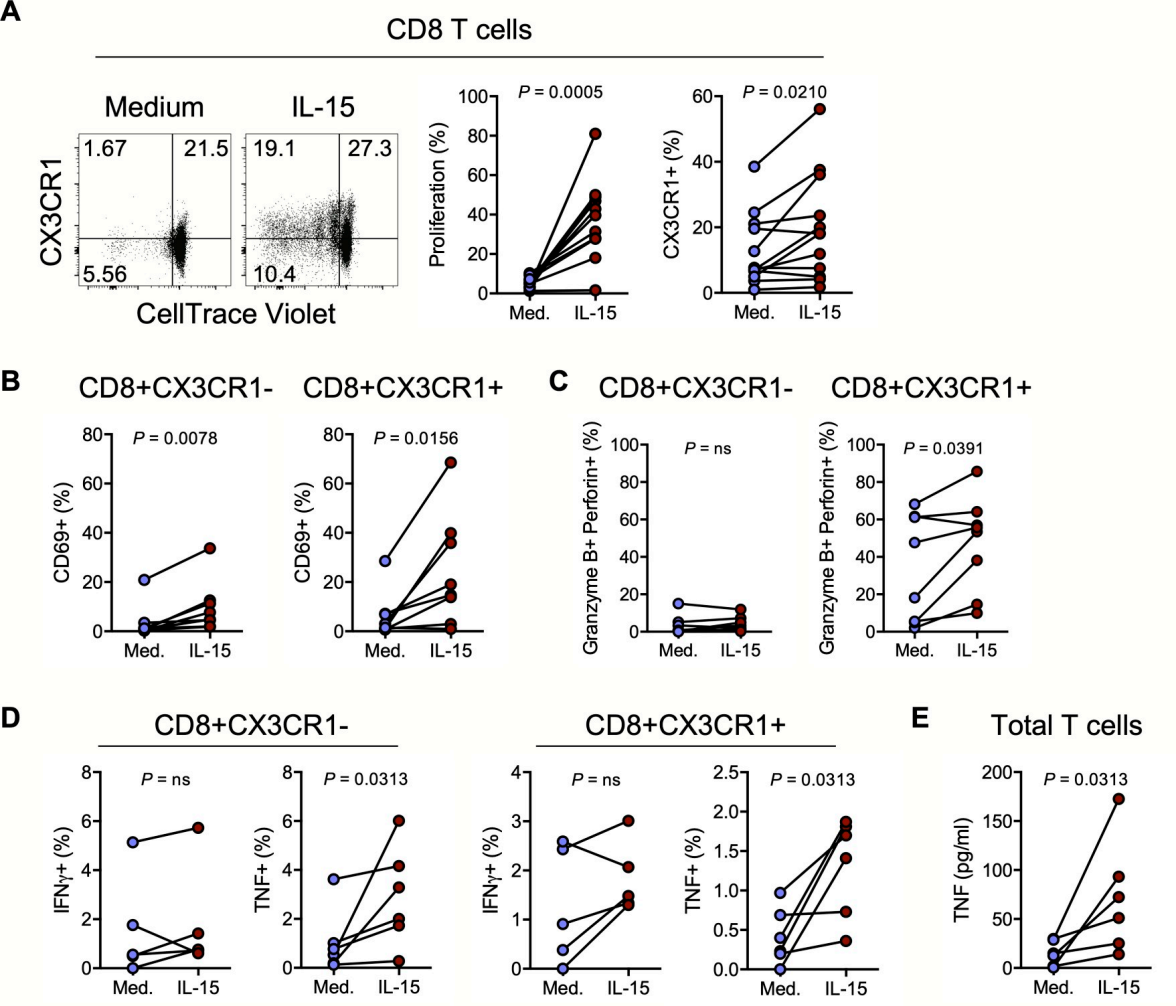
IL-15 drives CD8 T cell proliferation, activation, and an inflammatory, vascular homing, cytolytic phenotype. **(A)** Representative dotplots (n = 12) showing CX3CR1 and CellTrace Violet expression on CD8 T cells following exposure to IL-15 (20ng/ml) or medium control for 7 days (left), and summary data of proliferation (middle) and surface CX3CR1 expression (right). **(B)** Proportion of CX3CR1- CD8 T cells (left) and CX3CR1+ CD8 T cells (right) that express surface CD69 after 48h exposure to IL-15 or medium control (n = 8). **(C)** Proportion of CX3CR1- CD8 T cells (left) and CX3CR1+ CD8 T cells (right) that exhibit intracellular granzyme B and perforin co-expression after 48h exposure to IL-15 or medium control (n = 8). **(D)** Proportion of CX3CR1- CD8 T cells (left) or CX3CR1+ CD8 T cells (right) that express intracellular IFNγ (n = 5) and TNF (n = 6) following overnight exposure to IL-15 or medium control. **(E)** Purified T cell (n = 6) TNF production following 48h exposure to IL-15 or medium control as measured by ELISA. Differences between groups were determined by Wilcoxon rank sum test. Med., medium.

As CD8 T cells are proximate to IL-15-expressing endothelium in atherosclerotic plaques and in SIV/SHIV-infected RM aortas, we proposed that vascular-infiltrating CD8 T cells might be exposed to IL-15, and performed an *in vitro* transcriptomic analysis to specifically define CD8 T cell signatures of IL-15 exposure. As expected [36, 37], we found that *in vitro* IL-15 exposure results in a profound shift in gene expression in CD8 T cells (S3A Fig), with 3868 genes significantly differentially regulated at 24 hours (S3B Fig), including genes important for cell cycle control and cell survival, adhesion and chemotaxis, and effector functionality (S3C Fig). CD8 T cells exposed to IL-15 for 7 days readily proliferated and the proportion of cells expressing CX3CR1 increased (Fig 4A), plausibly increasing the likelihood of CD8 T cell interactions with endothelial CX3CL1. Exposure to IL-15 also increased CD69 expression on both CX3CR1- and CX3CR1+ CD8 T cells (Fig 4B). Cytolytic potential–as reflected by granzyme B and perforin co-expression–was significantly enriched in CX3CR1+ CD8 T cells but not in CX3CR1- CD8 T cells after IL-15 exposure for 48 hours (Fig 4C). Finally, and in accordance with our transcriptional analysis (S3C Fig), exposure of both CX3CR1- and CX3CR1+ CD8 T cells to IL-15 for 24 hours modestly increased intracellular IFNγ and significantly increased TNF (Fig 4D). The IL-15-induced increase in CD8 T cells expressing intracellular TNF was corroborated by an increase in secreted TNF found in the supernatant of T cells exposed to IL-15 (Fig 4E), supporting our model that IL-15 promotes endothelium-homing (via CX3CR1), tissue retention (via CD69), and proinflammatory function (e.g. TNF production) of CD8 T cells.

### TNF induces expression of CX3CL1 and IL-15 in primary human aortic endothelial cells

**Fig 5:**
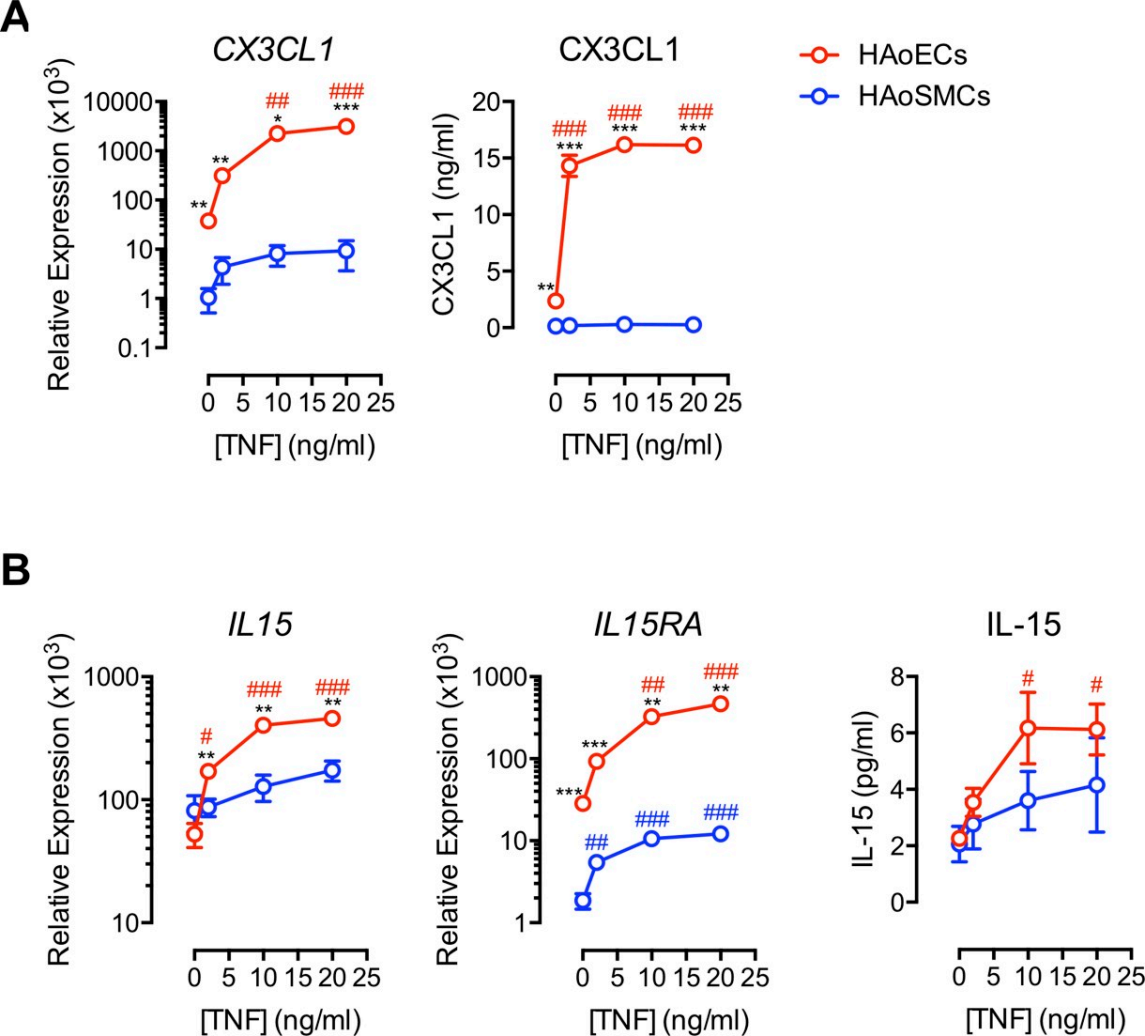
TNF induces primary HAoEC expression of CX3CL1 and IL-15. HAoECs and HAoSMCs were cultured for 7 days in the presence of TNF or medium control. **(A)** Relative *CX3CL1* expression compared to GAPDH (left) and CX3CL1 secretion (right) in HAoECs (n = 3) and HAoSMCs (n = 3). **(B)** Relative expression of *IL15* and *IL-15RA* compared to GAPDH (left and middle) and IL-15 secretion (right) in HAoECs (n = 3) and HAoSMCs (n = 3). Data are mean ± SEM. Differences between groups were determined by Mann-Whitney U test: *P ≤ 0.05; **P ≤ 0.01; ***P ≤ 0.001. Differences within groups were determined by Kruskal-Wallis test with Dunn’s correction: #*P* ≤ 0.05; ##*P* ≤ 0.01; ###*P* ≤ 0.001. HAoECs, human aortic endothelial cells; HAoSMCs, human aortic smooth muscle cells.

We next examined the effects of TNF on HAoECs and HAoSMCs to explore potential bidirectional interactions between endothelial-homing CD8 T cells and vascular tissues. TNF enhanced the expression and secretion of CX3CL1 by HAoECs, consistent with our recent reports [15, 33], but not by HAoSMCs (Fig 5A). TNF has also been shown to elicit IL-15 expression by human umbilical vein ECs [38], and similarly, we found that TNF could induce IL-15 secretion by both HAoECs and HAoSMCs. TNF-induced expression of both *IL15* and *IL15RA* was dramatic in HAoECs but only modest in HAoSMC (Fig 5B). Thus, we have identified a possible feed-forward model of atherosclerosis in which plaque-infiltrating CX3CR1+ CD8 T cells are exposed to vascular EC-derived IL-15 that induces antigen-independent TNF release, resulting in enhanced release of CX3CL1 and IL-15 from ECs. It is possible that cognate antigen recognition could also promote T cell activation in the vasculature during viral infection. We found SIV/SHIV Gag protein in the aortas of infected macaques (S1C Fig), and we have detected nucleic acids of other viruses within atherosclerotic plaque tissues of humans [39]. In additional to indirect proinflammatory effects, viruses also might directly alter endothelial function. Direct exposure of HAoECs to HIV particles resulted in endothelial activation evidenced by upregulation of vascular cellular adhesion molecule (VCAM)-1 and tissue factor (S4 Fig).

### Chemoattraction of activated CX3CR1+ CD8 T cells to an EC monolayer

**Fig 6:**
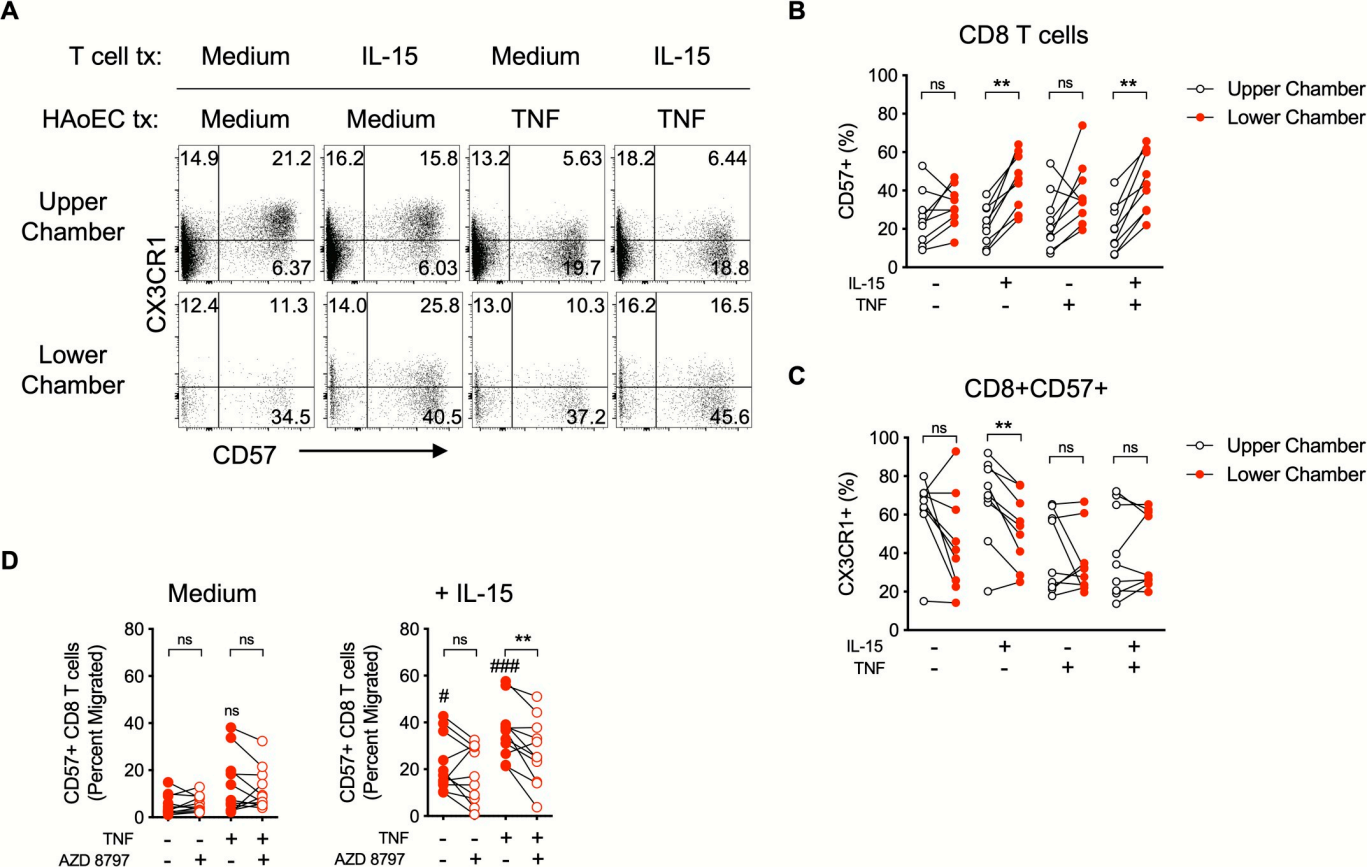
Chemoattraction of activated CX3CR1+ CD8 T cells to an endothelial cell monolayer. **(A)** Representative dotplots showing CX3CR1 and CD57 expression on CD8 T cells. **(B)** Percentage of CD8 T cells in each chamber expressing CD57. **(C)** Percentage of CD57+ CD8 T cells in each chamber expressing CX3CR1. **(D)** Absolute number of CD57+ CD8 T cells in the lower chamber expressed as a percent of total (upper chamber + lower chamber) CD57+ CD8 T cells (“Percent migrated”) pre-treated with medium control (left) or IL-15 (right). Actual numbers of cells recovered in the assay are depicted in S5 Fig. **(B-D)** Differences between groups were determined by Wilcoxon rank sum test: *P ≤ 0.05; ns, not significant. HAoECs, human aortic endothelial cells; tx, treatment.

To further test our model, we used an *ex vivo* transmigration assay system that we recently developed [33] to determine if dysfunctional endothelium could promote the migration of activated CX3CR1+ CD8 T cells. Because exposure to CX3CL1 downmodulates CX3CR1 expression (Fig 2G), we used CD57 on the surface of CD8 T cells as a marker to identify cells that are or were recently CX3CR1+ [32]. We found that a greater proportion of the CD8 T cells that migrated to the lower chamber were CD57+ (and thus were likely initially CX3CR1+) (Fig 6A and 6B). There was a significant reduction in the percentage of CD57+ CD8 T cells in the lower chamber that were CX3CR1+ when the T cells were pre-treated with IL-15 but not with medium control, and pre-treatment of HAoECs with TNF, which induces robust CX3CL1 expression (Fig 5A), decreased CX3CR1 expression on CD57+ CD8 T cells in both chambers (Fig 6A and 6C) suggesting that the CD8 T cell migration is linked to CX3CL1 ligation. Migration was particularly enhanced when the T cells were pre-treated with IL-15 (Fig 6D, S5 Fig), which promotes CX3CR1 expression (Fig 4A, S3C Fig) [32]. The CX3CR1 antagonist AZD8797 [40–42] significantly reduced the numbers of IL-15-primed CD57+ CD8 T cells that migrated across the membrane toward HAoECs pre-treated with TNF (Fig 6D). These data implicate CX3CR1/CX3CL1 interactions as driving migration of CD57+ CD8 T cells toward TNF-activated endothelium. Taken together, our findings indicate that IL-15 has a pro-migratory effect on CD8 T cells toward TNF-treated (CX3CL1-producing) HAoECs that is at least partially dependent on CX3CR1.

## Discussion

**Fig 7:**
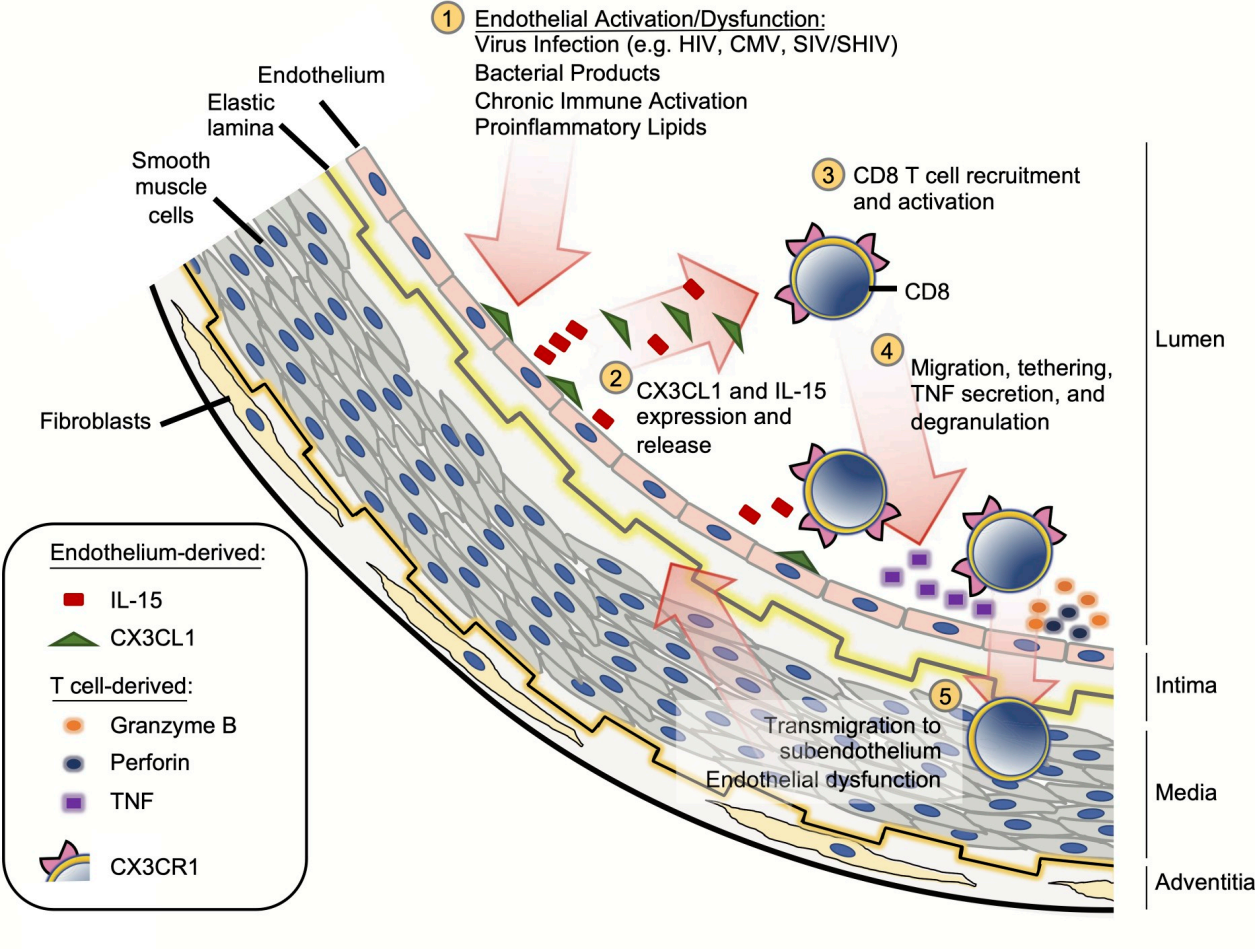
Model linking CX3CR1+ CD8 T cells, TNF, and IL-15 to endothelial dysfunction and vascular inflammation. **(1)** Endothelial cell activation/dysfunction is induced by viral infection, bacterial products, and/or proinflammatory cytokines and lipids; **(2)** Activated endothelial cells express and release CX3CL1 and IL-15; **(3)** IL-15 activates CD8 T cells by arming them with the cytolytic molecules, promoting CX3CR1 expression, and increasing TNF production; **(4)** CX3CL1/CX3CR1 interactions promote CD8 T cell migration and tethering to the activated endothelium, where the cells release TNF and lytic granules like granzyme B and perforin; **(5)** TNF and endothelial damage further increase CX3CL1 and bioactive IL-15 expression by vascular endothelial cells creating a positive proinflammatory feedback loop that facilitates continued endothelial cell activation, damage, and accumulation of activated CD8 T cells in the subendothelium. These changes may promote atherogenesis in HIV infection and in HIV-uninfected individuals with persistent vascular inflammation.

CVD is a major cause of morbidity and mortality in the general aged population.[43] Among PLWH, even those with complete viral suppression on ART experience earlier onset and increased prevalence of CVD [1–4], and as PLWH on ART age, cardiovascular comorbidities are likely to become an even more substantial burden. We sought to dissect the underlying mechanisms of atherosclerotic CVD by focusing on potential interactions between the vascular endothelium and activated CX3CR1+ CD8 T cells–cells that increase in prevalence with age (S2E Fig), and are increased dramatically in those with clinical CVD and in those with HIV infection [7, 8, 44]. First, we identified increased expression of CX3CL1 and IL-15 in the aortic endothelium of monkeys infected with SIV or SHIV and in carotid arteries and plaques of HIV-uninfected persons with atherosclerosis. In plaques, the proximity of CX3CL1 and IL-15 to infiltrating CD8 T cells, many of which are activated with evidence of CX3CL1 exposure and phenotypes compatible with IL-15 exposure, suggests the potential importance of these molecules in disease pathology. Second, we demonstrated that *in vitro*, primary HAoECs but not SMCs produce CX3CL1 and also produce both IL-15 and its alpha receptor that are each necessary for IL-15 bioactivity. Third, we demonstrated that IL-15-activated CD8 T cells upregulated expression of CX3CR1, cytolytic molecules, and TNF. TNF in turn activated HAoECs in culture to produce elevated levels of CX3CL1 and IL-15. Finally, we showed that CX3CR1/CX3CL1 interactions promote chemoattraction of IL-15-treated CD8 T cells toward TNF-treated ECs. These findings link disparate biological observations to a novel model of CD8 T cell contribution to atherosclerotic CVD that may be prevalent in aging individuals and enhanced in PLWH (Fig 7).

Our data confirm and build upon previous observations that IL-15 is expressed within atherosclerotic plaques [16, 17], that IL-15 promotes plaque development in a mouse model of atherosclerosis [18], and that polymorphisms in *IL15* are associated with subclinical atherosclerosis [45]. It has been postulated that plaque-infiltrating macrophages produce IL-15, and while we did not investigate macrophages here, our data clearly demonstrate that vascular ECs are capable of IL-15 production in culture, and that TNF, which can be expressed by infiltrating CD8 T cells, increases HAoEC IL-15 expression. We also found for the first time that HAoECs were capable of expressing IL-15Rα, which is necessary for the bioactive *trans*-presentation of IL-15 [34]. Our data are thus in line with Oppenheimer-Marks, et al., who showed that umbilical vein endothelial IL-15 could enhance the transendothelial migration of T cells [46], and with Liu, et al., who showed that TNF could induce IL-15 and IL-15Rα on the surface of endothelial cells [38]. Previously, elevated expression of CD25 has been observed on CD8 T cells recovered from plaques [14], consistent with *in vivo* exposure to IL-15, an interpretation supported by our observation of increased CD69 expression on plaque CD8 T cells. We also demonstrate here that HAoECs produced CX3CL1 *in vitro*, and exposure to a product of CD8 T cells (TNF) dramatically increased CX3CL1 gene expression and protein secretion. Polymorphisms in *CX3CR1* have been associated with altered CVD risk [9, 10], suggesting a role for CX3CR1/CX3CL1 interactions in its development [47, 48]. Accordingly, CX3CR1 expression on CD8 T cells, endothelial production of CX3CL1, and T cell CX3CL1-mediated adhesion and chemotaxis are all increased in people with coronary artery disease, and attenuated following six months of statin therapy, which decreases risk of CVD events [44]. In ART-treated HIV infection, we have shown CX3CR1+ CD8 T cell expansion is associated with a lower CD4/CD8 ratio [7] that is linked to cardiovascular morbidity and mortality [49].

Our data suggest a central role for TNF in endothelial dysfunction and vascular disease. This is consistent with the literature–TNF is upregulated in atherosclerosis [50, 51], is a major activator of endothelial cells *in vitro* [15, 33, 52–54], and a recent meta-analysis has found that pharmacological targeting of TNF appears to be associated with reduced cardiovascular events in patients with rheumatoid arthritis [55, 56]. In addition, plasma levels of soluble TNF receptor (sTNFR)-I and sTNFR-II, which are *in vivo* markers of TNF activity, highly predict the development of cardiovascular morbidities in PLWH [57].

We focus here on CD8 T cell and EC interactions. Nonetheless, additional intercellular interactions (among T cells, NK cells, ECs, monocytes/macrophages, and platelets) are important for the ultimate nature and severity of the vascular lesion [51, 58–60]. In our previous study of aortas from SIV/SHIV-infected RM, we found CD8 T cells in proximity to CD68+ macrophages at sites of endothelial dysfunction [13], and we have recently observed that activated CD8 T cell production of TNF induces monocyte surface expression of tissue factor [15], an initiator of the extrinsic coagulation cascade [61]. Earlier, we found that patrolling blood monocytes are enriched for CX3CR1 expression [6, 62] that targets them to CX3CL1-expressing endothelium, and show here that TNF treatment induces both HAoECs and HAoSMCs to secrete the monocyte/macrophage-chemoattractant protein CCL2 (S6 Fig). In addition, we recently demonstrated that thrombin, a late product of tissue factor-induced coagulation, can activate protease-activated receptor (PAR)-1-expressing CX3CR1+ CD8 T cells to release IFNγ, yet induces platelets to release TGF-β that attenuates this IFNγ release [7]. Thus, platelets may serve an underappreciated regulatory role in the vascular microenvironment.

The mechanisms that drive cardiovascular risk in ART-treated PLWH and in aged HIV-uninfected individuals are incompletely understood, and our data fill a gap in that knowledge. In HIV infection there is persistent systemic inflammation (attributed to low-level virus replication, coinfections, microbial translocation, and a proinflammatory lipid environment, among others) [63–65] that leads to EC activation and dysfunction, creating a microenvironment rich with CX3CL1, which attracts CX3CR1+ CD8 T cells to the endothelium. Whether direct recognition of viral antigens on infected cells in the vasculature wall or plaque contributes to the T cell activation and recruitment is unknown. However, here we show that IL-15 that is elaborated by endothelium promotes further activation and recruitment of T cells, triggering the antigen-independent release of TNF, which then perpetuates subendothelial inflammation in a feed-forward manner to further promote endothelial dysfunction, CX3CL1 release, and more T cell infiltration (Fig 7). CD57+ CD8 T cells, which are enriched for CX3CR1 expression and for expression of the cytolytic enzymes granzyme B and perforin, are plausible contributors to this process [32], and as we show here, migration of CD57+ CD8 T cells toward activated endothelial cells is at least partially CX3CL1-dependent. Granzyme B and perforin release by the activated T cells could also contribute to endothelial cell damage and death–indeed, in an adoptive transfer mouse model of atherosclerosis, transfer of CD8 T cells deficient in granzyme B, perforin, or TNF attenuated atherosclerotic lesions [66]. Therefore, we predict these mechanisms of T cell-induced endothelial damage are operative in atherosclerosis of HIV-uninfected persons and are accelerated in PLWH who live in a state of sustained chronic inflammation and have increased CX3CR1+ CD8 T cell numbers. Our model offers several therapeutic targets that may attenuate or even prevent atherosclerosis in PLWH and in the elderly general population.

## Supporting information

S1 Fig Control staining and p27/Gag expression in sections from rhesus macaque aortas. (TIF)

S2 Fig Expression of CX3CL1 and IL-15 in human atherosclerotic plaque tissue in proximity to CD8 T cells. (TIF)

S3 Fig IL-15 promotes robust transcriptional changes in CD8 T cells. (TIF)

S4 Fig HIV exposure induces VCAM-1 and tissue factor expression in primary HAoECs. (TIF)

S5 Fig Quantification of the chemoattraction of CD57+ CD8 T cells toward an endothelial cell monolayer. (TIF)

S6 Fig TNF induces primary HAoEC and HAoSMC expression of CCL2. (TIF)

S1 Table Participant characteristics. (TIF)

S2 Table Plaque donor characteristics. (TIF)
