# Supplementary figures and images for: CX3CL1 and IL-15 Promote CD8 T cell chemoattraction in HIV and in atherosclerosis
Source: PLoS Pathog. 2020 Sep 25;16(9):e1008885. doi: 10.1371/journal.ppat.1008885 (PMC7540902; doi:10.1371/journal.ppat.1008885)

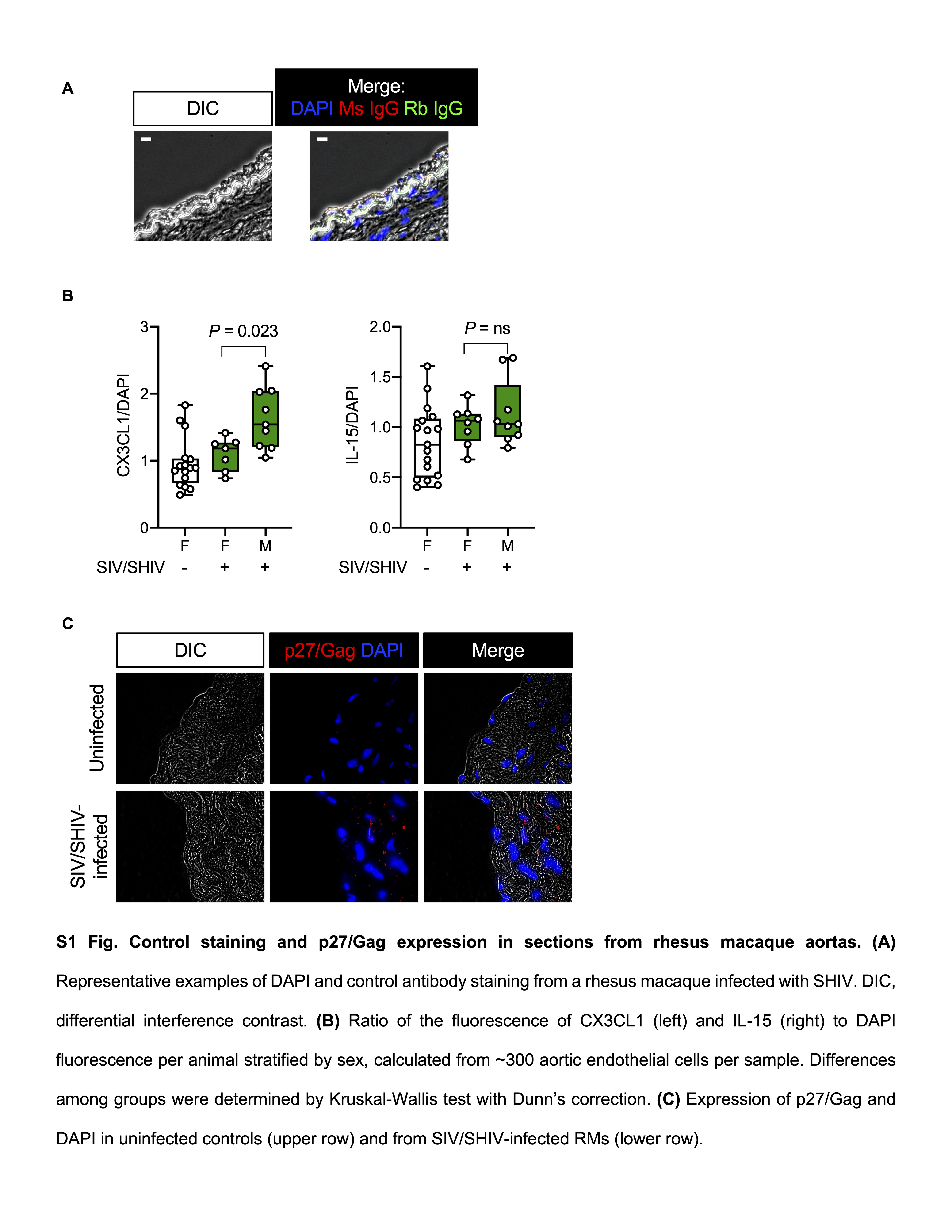

Supplement: S1 Fig — (TIF) [file ppat.1008885.s001.tif]

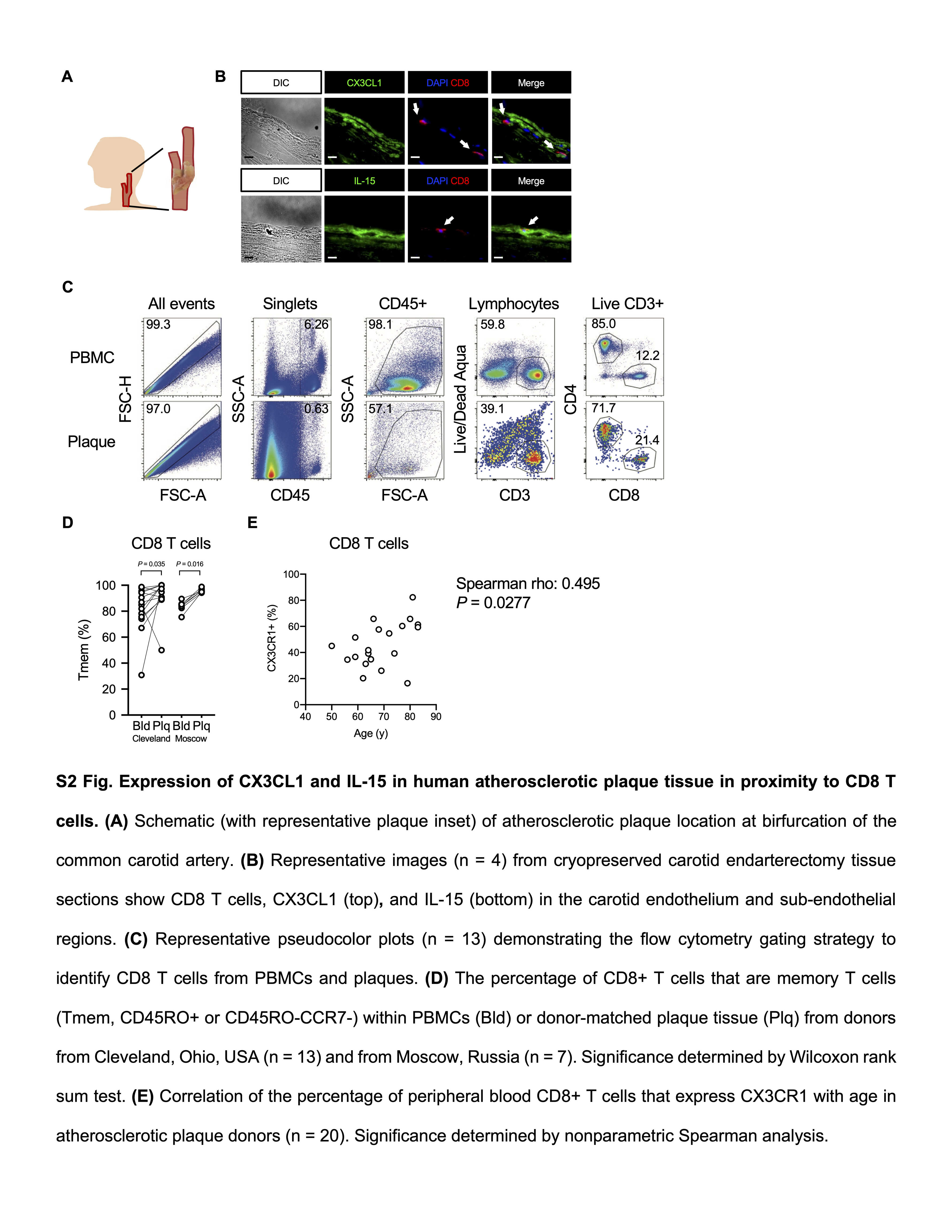

Supplement: S2 Fig — (TIF) [file ppat.1008885.s002.tif]

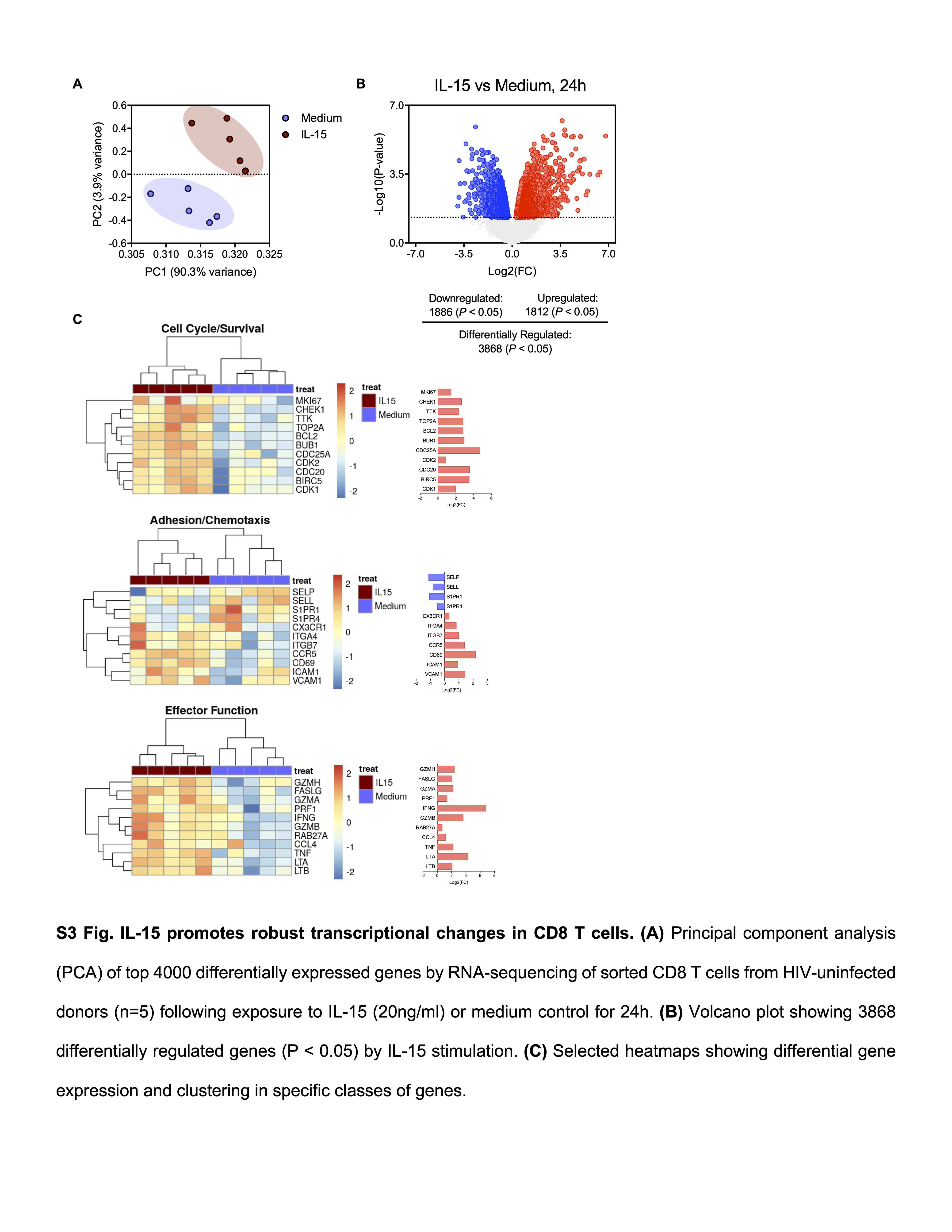

Supplement: S3 Fig — (TIF) [file ppat.1008885.s003.tif]

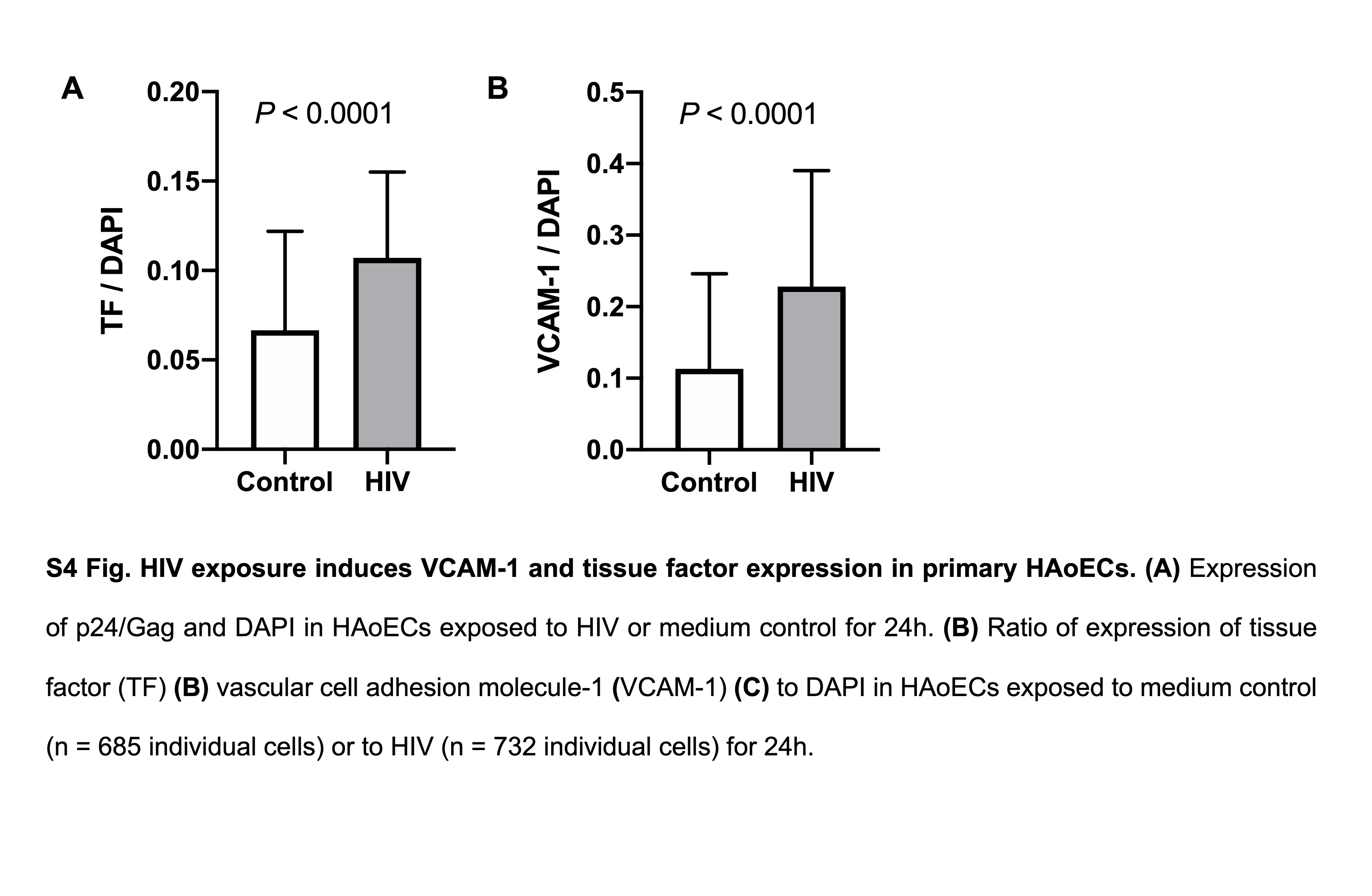

Supplement: S4 Fig — (TIF) [file ppat.1008885.s004.tif]

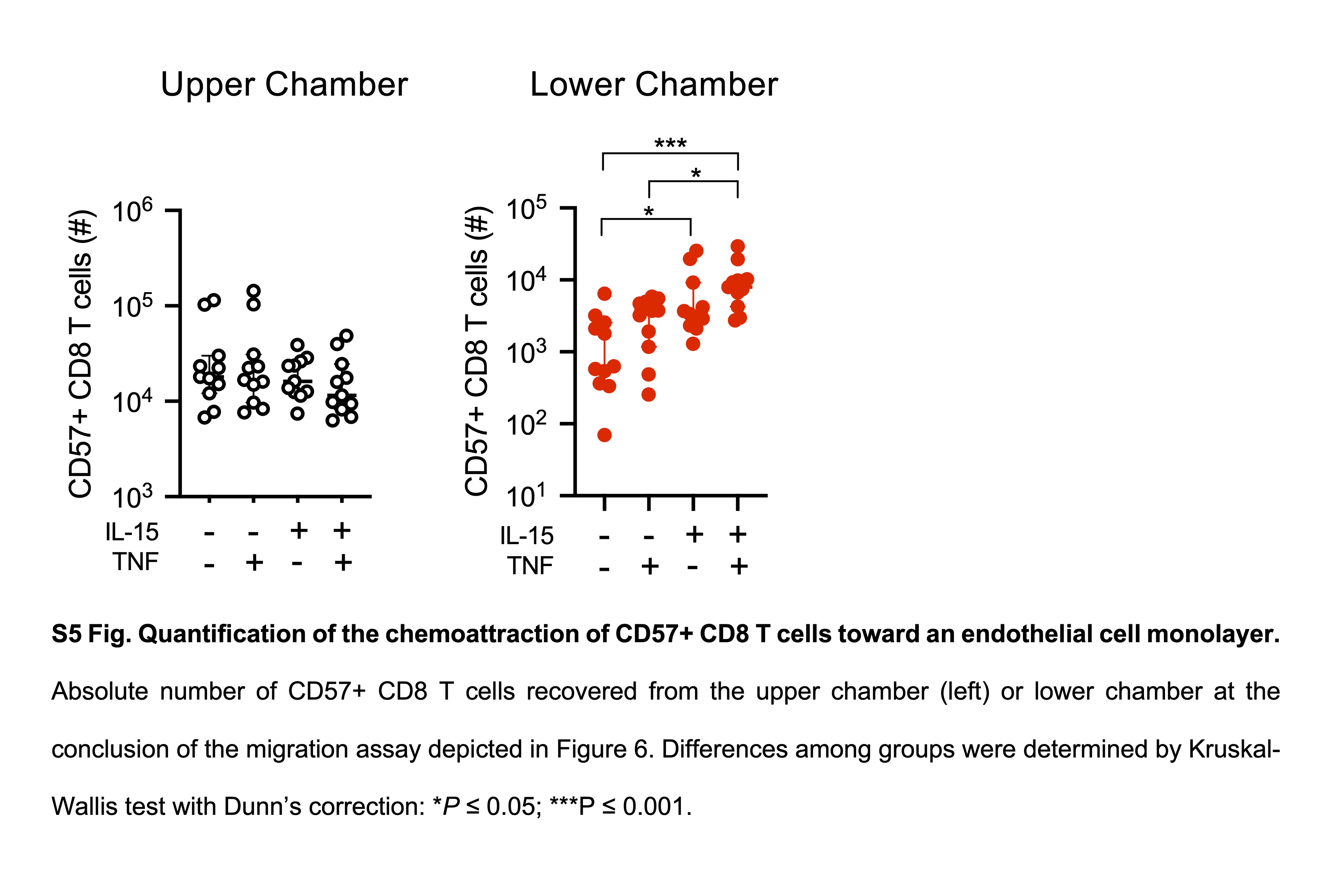

Supplement: S5 Fig — (TIF) [file ppat.1008885.s005.tif]

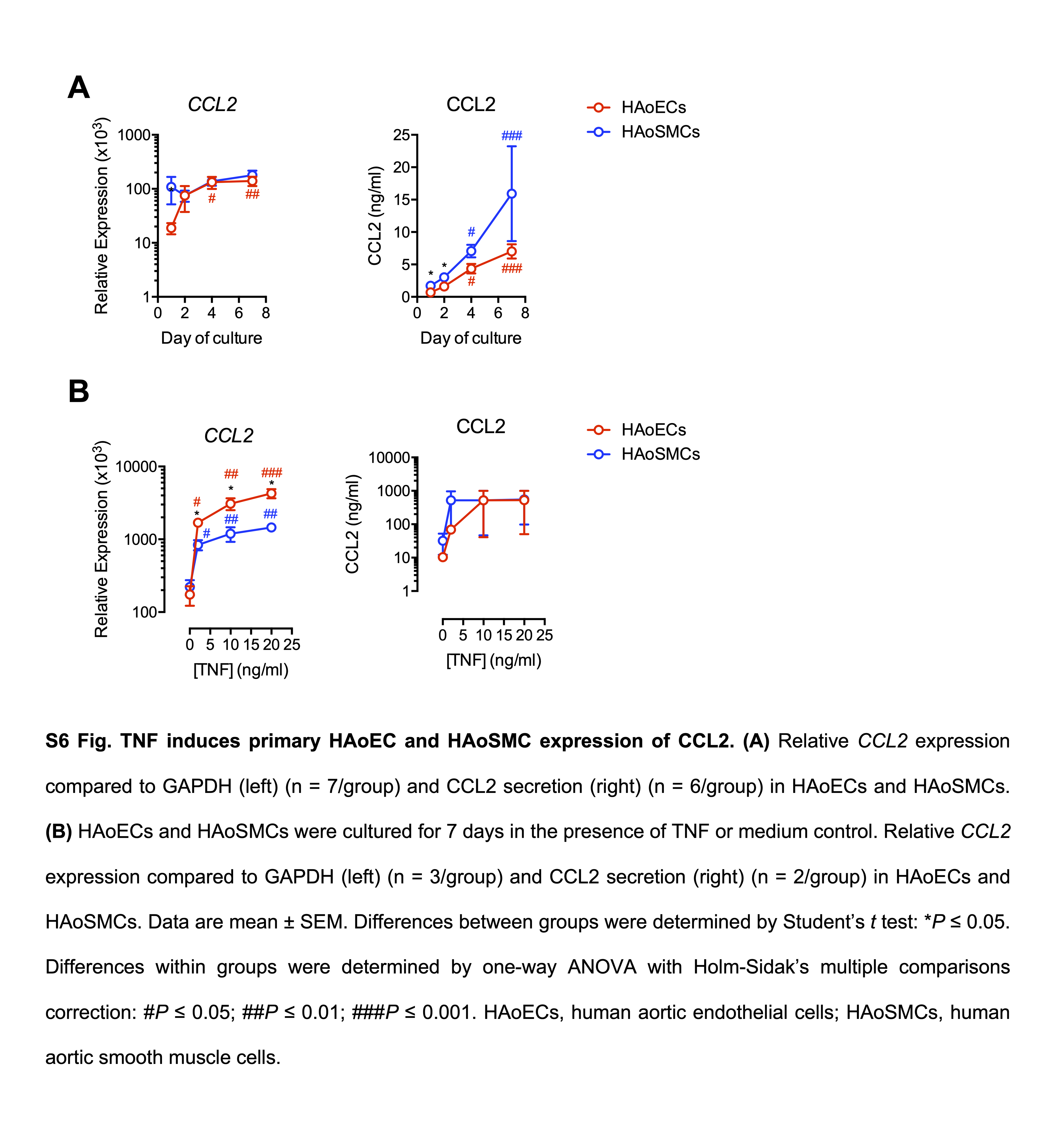

Supplement: S6 Fig — (TIF) [file ppat.1008885.s006.tif]

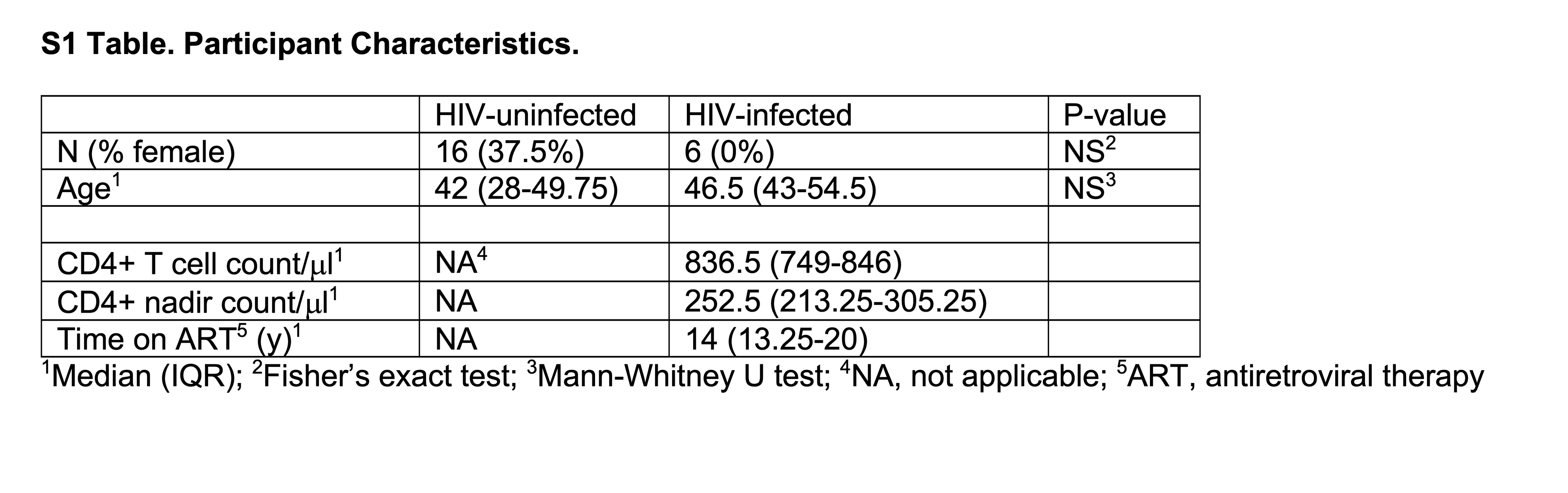

Supplement: S1 Table — (TIF) [file ppat.1008885.s007.tif]

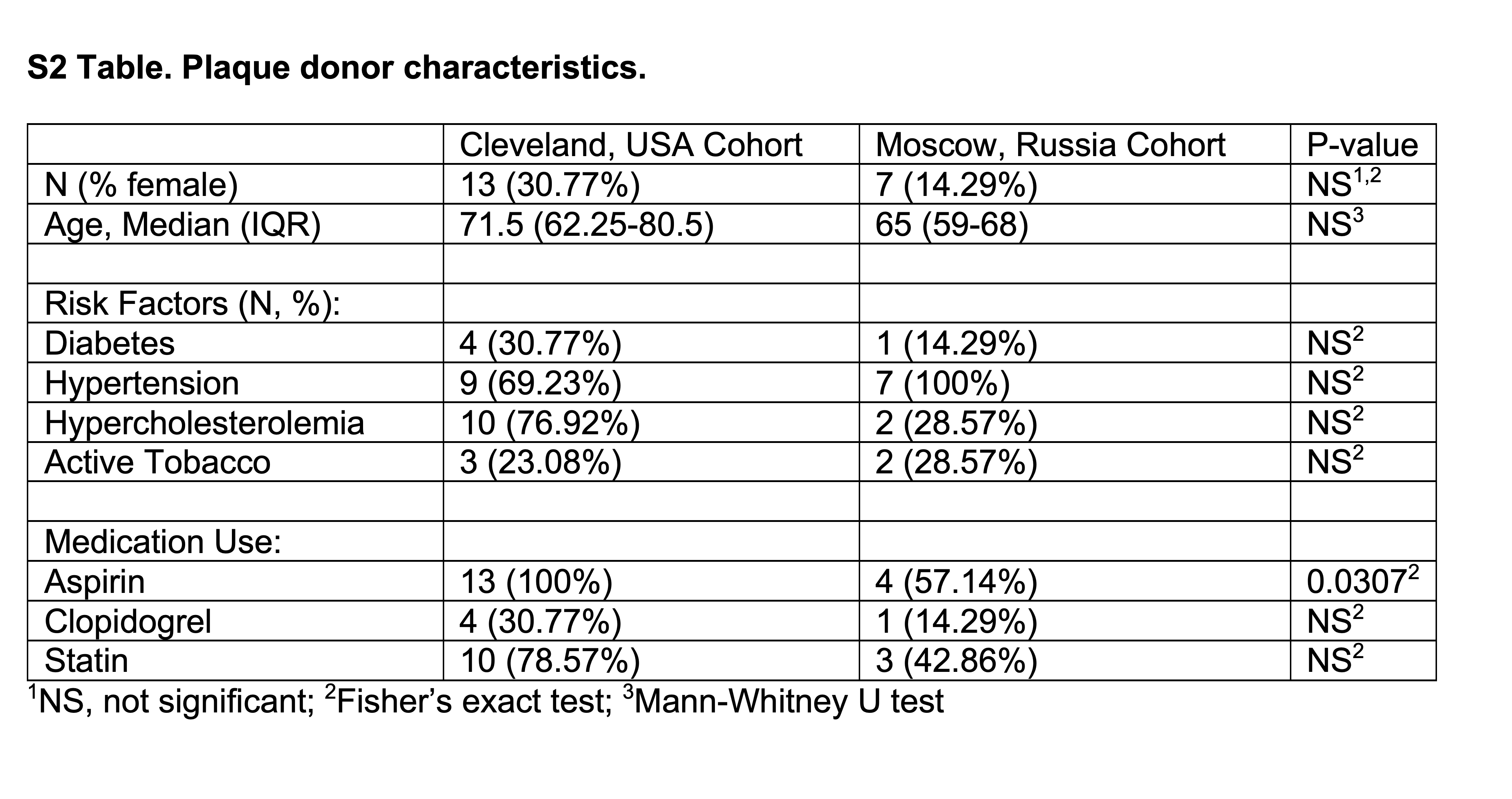

Supplement: S2 Table — (TIF) [file ppat.1008885.s008.tif]
